# Growth Response and Cell Permeability of the Fish-Killing Phytoflagellate *Heterosigma akashiwo* Under Projected Climate Conditions

**DOI:** 10.3390/toxins17050259

**Published:** 2025-05-21

**Authors:** Malihe Mehdizadeh Allaf, Charles G. Trick

**Affiliations:** 1 Department of Chemical and Biochemical Engineering, Western University, London, ON N6A 5B9, Canada; mmehdiz@uwo.ca; 2 Department of Physical and Environmental Sciences, University of Toronto, Toronto, ON M1C 1A4, Canada

**Keywords:** design of experiment (DOE), *Heterosigma akashiwo*, climate change, growth rate, cell permeability

## Abstract

Climate change and anthropogenic alterations in biogeochemical cycles are intensifying the frequency, duration, and potential toxicity of harmful algal blooms (HABs) in marine ecosystems. However, these effects are highly variable and depend on species identity, strain-specific traits, and local environmental conditions. Key drivers include rising sea surface temperatures, changes in salinity resulting from altered precipitation patterns and runoff, and elevated CO_2_ levels leading to ocean acidification. *Heterosigma akashiwo*, a euryhaline raphidophyte responsible for the widespread killing of fish, is particularly responsive to these changes. This study investigated the combined effects of temperature, salinity, and CO_2_ concentration on the growth, yield, and cell membrane permeability of *H. akashiwo* using a Design of Experiment (DOE) approach. DOE facilitates a detailed and systematic analysis of multifactorial interactions, enabling a deeper understanding of complex relationships while maximizing efficiency and minimizing the use of experimental resources. The results revealed that growth and yield were maximized at higher temperatures and salinities, whereas cell permeability increased under cooler, less saline, and lower CO_2_ conditions. These findings suggest that projected future ocean conditions may enhance biomass production while potentially reducing cellular permeability and, by extension, toxicity. This study highlights the value of the DOE framework in identifying key interactions among environmental drivers of HABs, offering a practical foundation for future predictive modeling under climate change scenarios.

## 1. Introduction

Harmful algal blooms (HABs) have been increasingly reported over recent decades, with evidence linking their frequency and intensity to climate change and accelerated eutrophication driven by domestic, industrial, and agricultural runoff [1,2,3,4,5,6]. These blooms encompass a broad diversity of phytoplankton species that vary in toxicity and geographic range. While HABs are often localized events, many causative species have global distributions. Blooms are characterized by excessive biomass accumulation, frequently in combination with the intracellular production of potent toxins [7]. Given the diversity of HAB-forming species, their responses to environmental change are not always predictable: toxicity can vary within and between species, and climate-driven shifts may favor the rise in novel or invasive taxa.

HABs pose serious ecological and socioeconomic threats, particularly in coastal systems. They can compromise public health via shellfish poisoning and respiratory irritation, disrupt fisheries and aquaculture, and degrade water quality [8,9,10,11,12]. In Canadian coastal waters, historical red tide events on the Pacific and Atlantic coasts suggest that rising atmospheric and oceanic temperatures are the key to bloom dynamics [13]. These temperature increases are extending the growing season [5,6], altering bloom phenology [14,15,16], and influencing phytoplankton traits such as growth [14], motility [17], and life cycle transitions [18].

Climate change also affects salinity through evaporation, precipitation, and freshwater runoff changes, particularly in coastal and estuarine systems [6,19,20,21,22,23,24,25,26,27]. Intensification of the hydrologic cycle [20,21] increases the influx of freshwater into marine systems, leading to significant spatial and temporal variability in salinity, a critical stressor for marine phytoplankton [22,23,28].

One of the most profound changes in marine environments is the rise in atmospheric CO_2_ concentrations, from pre-industrial levels of 280 ppm to over 426 ppm as of June 2024 [29]. Approximately half of this CO_2_ is absorbed by oceans [30,31], leading to the acidification of surface waters [32,33]. The resultant decrease in pH can disrupt cellular processes in phytoplankton, including enzyme activity, transmembrane potential, nutrient uptake [34,35,36,37,38,39,40], motility [39], and even ichthyotoxicity [38].

Understanding species-specific impacts is critical, given the complex interplay between climate change and phytoplankton responses. *Heterosigma akashiwo*, a euryhaline raphidophyte, has emerged as a dominant HAB species in many coastal regions. Its blooms are often associated with mass fish mortality [41,42,43]. The organism transitions between vegetative cells and benthic resting cysts, activated by temperature and light conditions [43,44,45,46]. Previous studies have shown that its growth is stimulated by temperatures exceeding 20 °C and CO_2_ concentrations above 700 ppm, which are the levels projected for the end of the 21st century [32,46,47,48,49,50,51,52,53].

Moreover, *H. akashiwo* exhibits a high tolerance to salinity variability, enabling it to thrive under fluctuating estuarine conditions [43,50,54,55,56]. Alteration in membrane permeability in response to salinity may represent a stress mitigation strategy to maintain osmotic balance [50]. For example, major blooms have occurred in English Bay, Vancouver, Canada, at 15 °C and low salinity (15) following snowmelt-driven runoff [57]. Despite the recognized influence of temperature, salinity, and CO_2_, three major environmental stressors associated with climate change, on *H. akashiwo*, few studies have assessed the combined effects of these factors. This study addresses this gap using a Design of Experiment (DOE) approach to systematically examine the interactions between temperature, salinity, and CO_2_ on the growth, yield, and membrane permeability of *H. akashiwo* [51,58]. Compared to the traditional one-factor-at-a-time (OFAT) approach, DOE provides a more robust statistical framework that simultaneously evaluates multiple factors and their interactions while significantly minimizing the number of experimental runs required [51,58,59,60]. This approach enhances efficiency and improves the reliability and interpretability of the results, particularly when investigating complex, multifactorial environmental stressors such as those associated with climate change. To our knowledge, this is the first study to apply DOE to identify the optimal ecological conditions for *H. akashiwo*’s performance. The findings enhance our predictive understanding of HAB dynamics in a rapidly changing ocean.

## 2. Results

### 2.1. H. akashiwo Growth Rates

**Figure 1:**
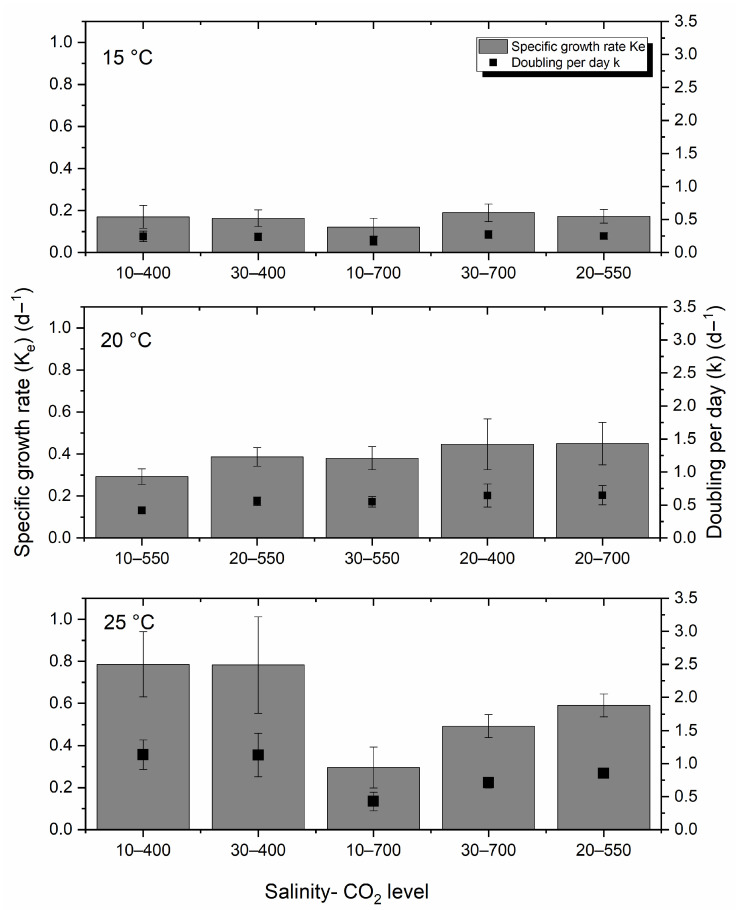
Profiles of specific growth rate (K_e_) and doublings per day (k) for *H. akashiwo* at 25 °C, 20 °C, and 15 °C at different salinities and CO_2_ levels. The bar chart represents the specific growth rate (K_e_) (d^−1^), and the scatter graph (■) illustrates the doublings per day (k) (d^−1^). The discrete data points are the average of the triplicate measurements ± standard deviation (*n* = 3).

Profiles of the specific growth rate (K_e_) and doublings per day (k) for *H. akashiwo* at three different temperatures of 25 °C, 20 °C, and 15 °C under varying salinities and CO_2_ levels are presented in Figure 1. Growth rate increased with temperature, with the highest values observed at 25 °C. Higher CO_2_ levels were associated with a decline in both K_e_ and k at different salinities at this temperature. In contrast, growth rates at 20 °C were relatively stable across CO_2_ and salinity gradients. Cultures at 15 °C showed the lowest growth and doubling rates, with no statistically significant variation across treatments.

After running a one-way analysis of variance (ANOVA) followed by a Tukey post hoc test, a significant difference was observed between 15 °C and 20 °C, and 15 °C and 25 °C, with *p*-values of 0.001 and 0.003, respectively, for the specific growth rate and doublings per day. In comparison, no significant difference was observed between 20 and 25 °C (*p*-value = 0.92).

#### 2.1.1. Response Surface Modeling Validation for Growth Rate

A total of 19 experiments were conducted using a three-factor, two-level, complete factorial design with center points (Table 1).

ANOVA results (Table 2) indicated that the models for both K_e_ and k were significant (*F*-value = 31.76 and *p*-value < 0.0001) and best fit by a two-factor interaction (2FI) model. Temperature, CO_2_ level, and the interaction between both parameters were significant main effects, with a *p*-value of <0.0001 and 0.0010 for growth rate and doublings per day, respectively. Salinity and other interactions (X_1_X_2_ and X_2_X_3_) were not statistically significant.

The model’s goodness of fit was defined by the coefficient of determination R-squared (0.9454) and the adjusted determination coefficient Adj. R-squared (0.9157) [61]. The adequate precision ratio of 17.80 further confirmed the model’s robustness.

After eliminating insignificant parameters, the model for the growth rate and doublings per day of *H. akashiwo* in terms of actual factors was as follows:

Specific growth rate (K_e_) = −1.25 + 0.105 × Temperature + 1.53 × 10^−3^ × CO_2_ level − 1.26 × 10^−4^ × Temperature × CO_2_ level

Doublings per day (k) = −1.80 + 0.152 × Temperature + 2.208 × 10^−3^ × CO_2_ level − 1.82 × 10^−4^ × Temperature × CO_2_ level

**Figure 2:**
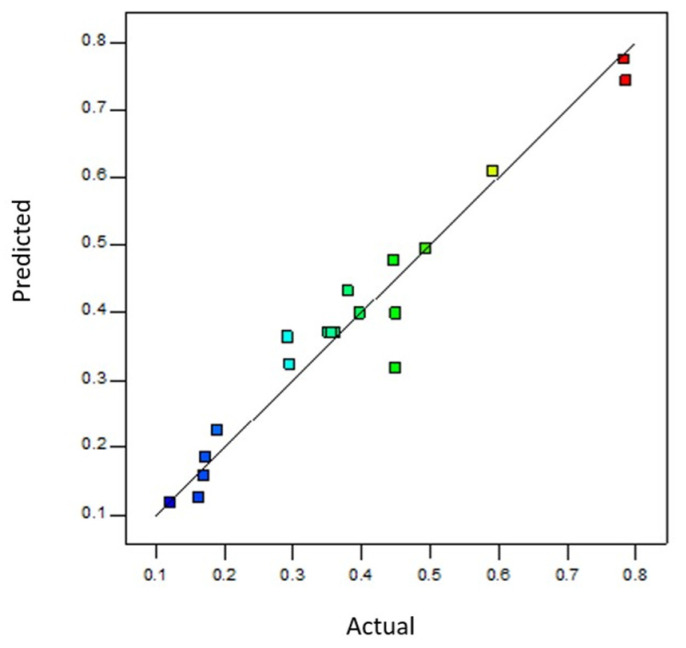
Comparison of actual and predicted values of specific growth rates (K_e_).

Based on the mathematical model obtained, temperature and CO_2_ level significantly positively affected growth rate and doublings per day. In contrast, the interaction between both factors had an adverse effect on similar responses. Figure 2 shows the strong agreement between the predicted and observed values, with residuals randomly distributed around the 1:1 line.

#### 2.1.2. Influence of Environmental Factors and Their Interactions on the Growth Rate

**Figure 3:**
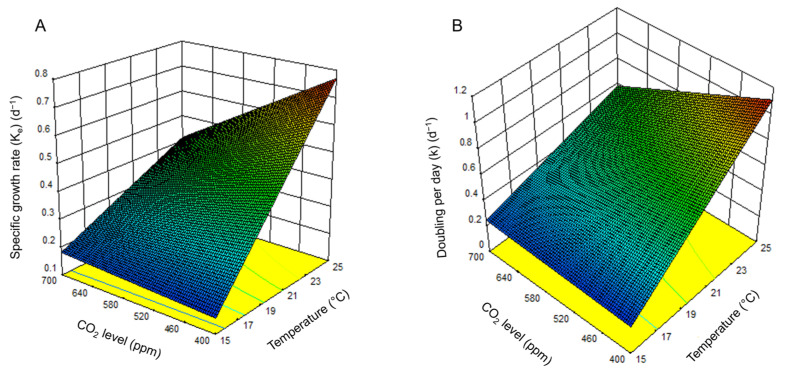
Surface plot of the combined effect of temperature and CO_2_ level on *H. akashiwo*. (**A**) Specific growth rate (K_e_). (**B**) Doublings per day (k).

Response surface plots (Figure 3A,B) revealed that temperature had a more substantial influence than CO_2_ on both K_e_ and k. However, their interaction significantly shaped the response surfaces, with optimal growth occurring at elevated temperatures and moderate CO_2_ levels.

Experimental validation confirmed the model predictions (Table 3), and a *t*-test at 95% confidence found no significant difference between the predicted and observed outcomes.

### 2.2. H. akashiwo Cell Yield

**Figure 4:**
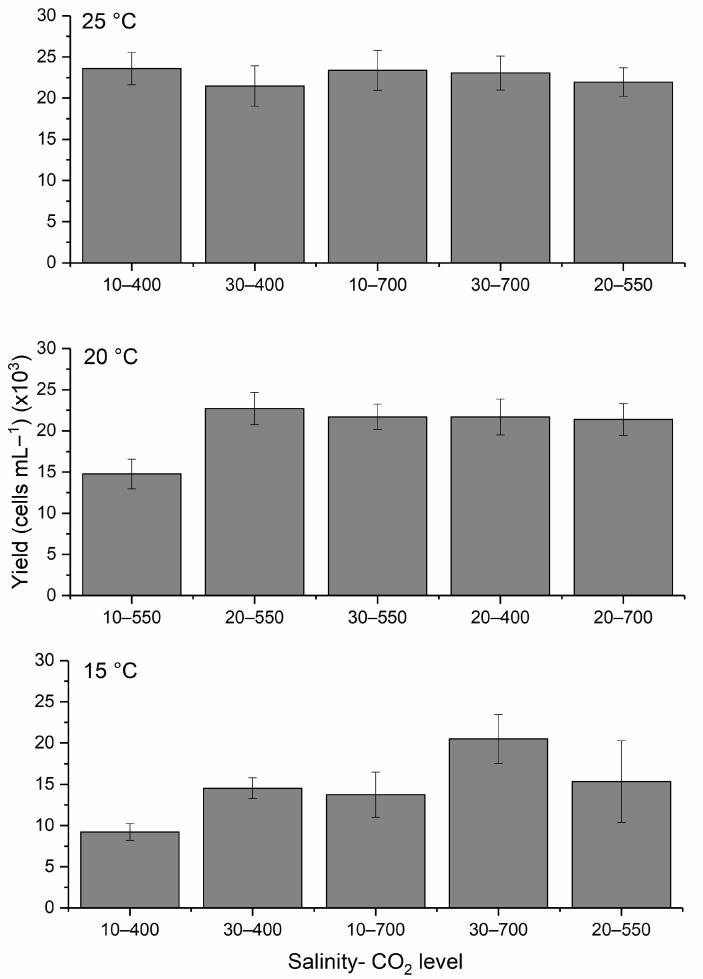
Yield of *H. akashiwo* grown at 25 °C, 20 °C, and 15 °C with different salinities and CO_2_ levels. The discrete data points are the average of triplicate measurements ± standard deviation (*n* = 3).

The highest yield (~23,000 cells mL^−1^) was observed at 20 °C, a salinity of 20 and a CO_2_ level of 550 ppm, which was followed by cultures grown at 25 °C; salinities of 10, 10, and 30; and CO_2_ levels of 400, 700, and 700 ppm, respectively (Figure 4). In contrast, the lowest cell yield occurred at the lowest temperature, salinity, and CO_2_ conditions (Figure 4). Warmer temperatures consistently supported higher cell yields. One-way ANOVA with Tukey post hoc analysis revealed a significant difference between 15 and 25 °C (*p*-value = 0.004) and 15 and 20 °C (*p*-value = 0.025), but not between 20 and 25 °C (*p*-value = 0.56).

#### 2.2.1. Response Surface Modeling Validation for Yield

A three-factor, two-level, complete factorial design with center points was applied to model cell yield under varying environmental conditions. Table 4 presents the design and results. Temperature significantly affected the yield production of *H. akashiwo* in this experiment. Increasing the temperature from 15 °C up to 25 °C, when media salinity was 10 and CO_2_ concentration was at the lowest level in this experiment, improved the biomass production and yielded more than two times the amount.

ANOVA (Table 5) confirmed that temperature was a statistically significant predictor of cell yield (*p*-value = 0.0005), with the model showing overall significance (*F*-value = 6.5 and *p*-value = 0.0093). Other factors (salinity and CO_2_) were less influential. To validate the adequacy of model fit, lack of fit, which is the variation in the data around the fitted model, was used [61,62]. A non-significant lack of fit (*F* = 5.18) confirmed model adequacy. The yield model, in terms of actual factors, was expressed by the following equation:

Yield = −61,315.03 + 5652.08 × Temperature

This positive linear relationship suggested that increasing temperature drives higher biomass production under the tested conditions.

**Table 5:** ANOVA results for the yield production of *H. akashiwo*.

Source	Remark	Sum of Squares	df	Mean Square	*F*-Value	*p*-Value
Model	Significant	2.842 × 10^8^	9	3.158 × 10^7^	6.05	0.0093
X_1_	Significant	1.609 × 10^8^	1	1.609 × 10^8^	30.85	0.0005
X_2_		2.745 × 10^7^	1	2.745 × 10^7^	5.26	0.0510
X_3_		1.332 × 10^7^	1	1.322 × 10^7^	2.55	0.1487
X_1_X_2_		2.631 × 10^7^	1	2.631 × 10^7^	5.04	0.0549
X_1_X_3_		1.032 × 10^7^	1	1.032 × 10^7^	1.98	0.1972
X_2_X_3_		1.289 × 10^6^	1	1.289 × 10^6^	0.25	0.6325
X_1_^2^		1.112 × 10^7^	1	1.112 × 10^7^	2.13	0.1824
X_2_^2^		1.586 × 10^7^	1	1.5886 × 10^7^	3.04	0.1193
X_3_^2^		1.874 × 10^6^	1	1.874 × 10^6^	0.36	0.5655
R-squared						0.8720
Adj. R-squared						0.7280
Adeq precision						8.383

#### 2.2.2. Main and Interaction Influence of Factors on Yield

**Figure 5:**
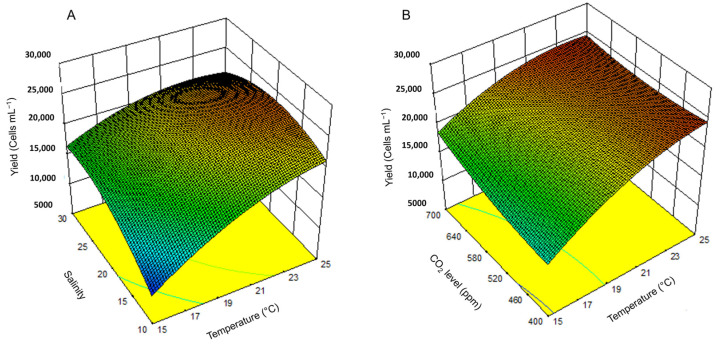
Surface plot of the combined effect of temperature and salinity (**A**) and temperature and CO_2_ level (**B**) on the cell yield of *H. akashiwo*.

Figure 5 illustrates the interaction effects of temperature with salinity and CO_2_. The lowest yields were associated with low temperature and salinity. In contrast, the highest yields were observed at 25 °C, a salinity of 20, and a CO_2_ level of 700 ppm. The model predicted a maximum yield of 24,746.1 ± 2283.81 cells mL^−1^ with a 95% prediction interval of 2311 to 30,078 cells mL^−1^. Experimental results (2511 ± 1052 cells mL^−1^) matched the predictions closely, with no significant difference (*t*-test, *p*-value > 0.05).

### 2.3. H. akashiwo Cell Permeability

**Figure 6:**
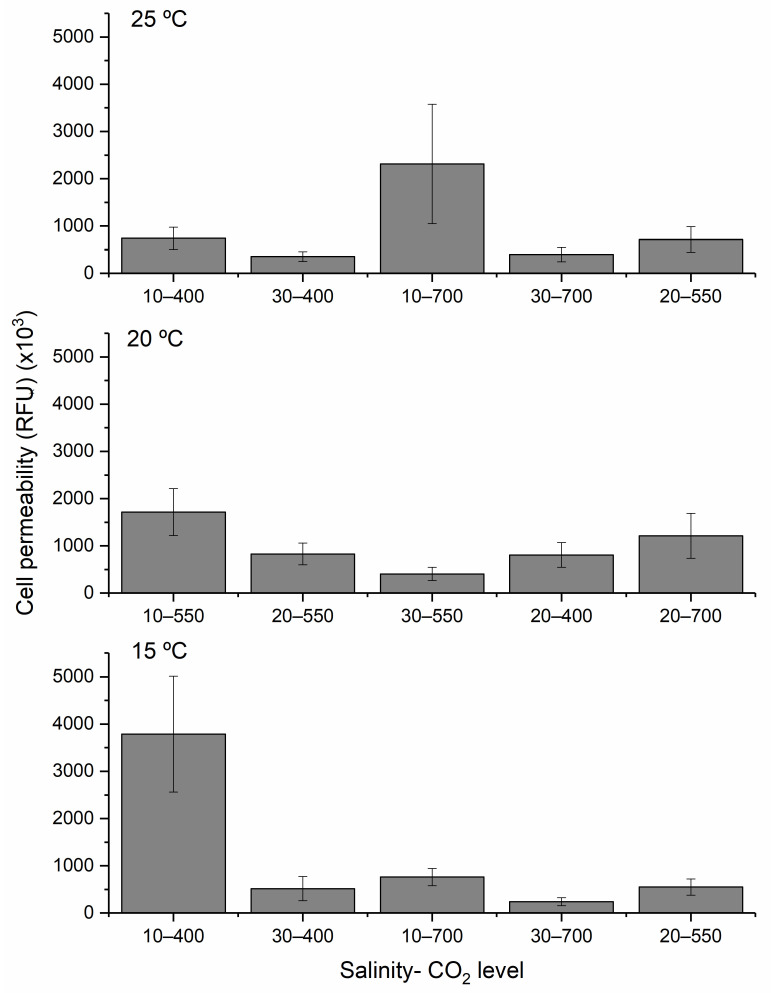
Cell permeability of *H. akashiwo* grew at 25 °C, 20 °C, and 15 °C with different salinities and CO_2_ levels. The discrete data points are the average of triplicate measurements ± standard deviation (*n* = 3).

Maximum membrane permeability was observed at the lowest temperature (15 °C), salinity (10), and CO_2_ level (400 ppm) (Figure 6). Salinity had the most decisive influence: decreasing salinity significantly increased membrane permeability. Permeability was lowest at the highest salinity level tested. While temperature had no significant effect (*p*-value > 0.05), both salinity and CO_2_ levels showed substantial effects (*p*-value < 0.05).

#### 2.3.1. Response Surface Modeling Validation for Cellular Permeability

Table 6 and Table 7 provide the design matrix and ANOVA results. The model was significant (*F*-value indicating only a 0.93% chance due to noise). Adequate precision (9.4) confirmed the model’s reliability. Salinity (*p*-values = 0.0013) and the interaction between temperature and CO_2_ (*p*-values = 0.0089) were significant factors.

The fitted 2FI polynomial model in terms of the significant actual factors is as follows:

Cell permeability = +1.51 × 10^7^ − 2.05 × 10^5^ × Salinity + 819.97 × Temperature × CO_2_ level

This indicates that salinity negatively impacts the cell permeability of *H. akashiwo,* while the interaction effect of temperature and CO_2_ level has a positively impact on the same response.

#### 2.3.2. Influence of Environmental Factors and Their Interactions on Cell Permeability

**Figure 7:**
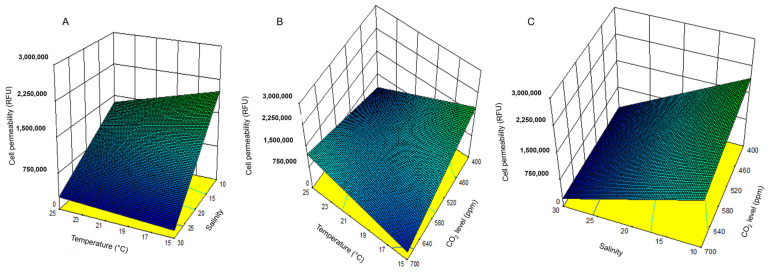
The combined effect of temperature and salinity (**A**), temperature and CO_2_ level (**B**), and salinity and CO_2_ level (**C**) on the cellular permeability of *H. akashiwo*.

Three-dimensional response surface graphs (Figure 7) illustrate temperature, salinity, and CO_2_ interactions. Permeability increased with decreasing salinity (Figure 7A,C) and lower temperature and CO_2_ levels (Figure 7B). The highest observed permeability (3,790,029 ± 1,226,958 RFU) was at 15 °C, a salinity of 10, and a CO_2_ of 400 ppm—closely aligning with model predictions.

### 2.4. Relationship Between Different Responses

**Figure 8:**
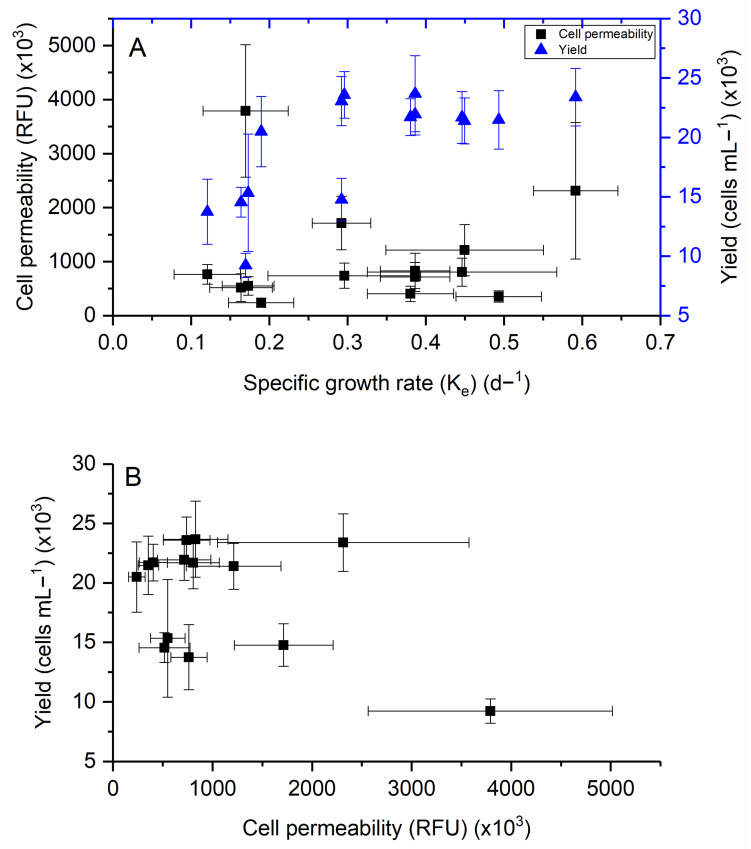
Effect of cell permeability and cell yield as a function of specific growth rates (**A**), and the relationship between cell permeability and cell yield production (**B**) in *H. akashiwo*.

The relationships among specific growth rate, yield, and cell permeability were examined to assess potential trade-offs and interdependencies. Most samples with varying growth rates exhibited cell permeability values below 2 ×10^6^ RFU (Figure 8A). Linear regression analyses indicated no significant relationship between growth rate and cell permeability (R^2^ ≈ 0), and a second-order polynomial regression also failed to reveal a meaningful association. Notably, cultures grown under the lowest temperature, salinity, and CO₂ conditions displayed the lowest growth rates but the highest cell permeability values.

In contrast, a positive linear relationship was observed between specific growth rate and cell yield (R^2^ = 0.61), indicating that faster growing populations generally achieved higher biomass (Figure 8A). Conversely, a negative relationship was found between yield and cell permeability (R^2^ = 0.57), suggesting that higher biomass production was associated with more stable, less permeable membranes (Figure 8B). Cultures with the most significant yield exhibited the lowest levels of membrane permeability.

These results highlight a potential trade-off between cellular integrity and productivity under environmental stress. While elevated temperature and salinity support growth and yield, they may suppress membrane permeability responses that are typically elevated under more stressful conditions.

## 3. Discussion

Despite increasing evidence of HABs under changing climate conditions, our understanding of how specific HAB species respond to simultaneous environmental stressors remains limited. Most studies adopt an OFAT approach, which fails to capture the interactive effects of multiple drivers that more accurately reflect natural conditions. In contrast, our study demonstrates the advantages of using a DOE methodology to explore how *H. akashiwo* responds to combined changes in temperature, salinity, and CO_2_ concentration [59]. The DOE approach comprehensively evaluated multiple stressors and revealed distinct growth, yield, and cell permeability patterns. The results indicated that warmer temperatures and elevated salinities promote higher growth rates and greater biomass production. This is consistent with earlier findings that *H. akashiwo* thrives in warm, high-salinity waters [47,48,49,50,51,63,64,65,66]. Moreover, the study confirmed that elevated CO_2_ levels (up to 700 ppm) enhanced growth when paired with optimal temperature conditions, supporting projections that *H. akashiwo* may benefit from climate-induced ocean acidification [48,52,53].

Cell permeability, interpreted here as a proxy for physiological stress, showed an inverse pattern. The highest permeability was recorded under the lowest temperature, salinity, and CO_2_ conditions, environments that inhibited growth and yield. This suggests that *H. akashiwo* exhibits stress-related membrane responses under suboptimal conditions, potentially contributing to its ichthyotoxicity in colder, fresher, and less buffered waters. Under stress conditions, in particular, some strains of *H. akashiwo* are known to release ichthyotoxic metabolites such as reactive oxygen species (ROS), including superoxide and hydrogen peroxide, as well as other bioactive compounds that may disrupt gill function in fish [43].

Notably, this study found no linear relationship between growth rate and cell permeability, implying that physiological stress and productivity are not necessarily coupled traits. However, a negative correlation between yield and cell permeability suggests a trade-off: conditions that promote biomass accumulation may suppress stress-induced permeability responses. This reinforces the idea that HAB toxicity cannot be predicted solely by cell density and must also consider physiological responses to environmental conditions.

By identifying optimal and suboptimal combinations of temperature, salinity, and CO_2_, this study provides insight into the ecological niche of *H. akashiwo.* These findings are particularly relevant for aquaculture risk management, bloom forecasting, and ecosystem health assessments.

## 4. Conclusions

DOE analysis is a powerful tool for predicting bloom dynamics in future oceans. This study demonstrates that the DOE framework can effectively identify the environmental conditions that optimize growth, yield, and cell membrane permeability in *H. akashiwo*. Key findings include the following: (1) Maximum growth rates occurred at 25 °C, a salinity of 30, and a CO_2_ of 400 ppm, with temperature being the most influential factor. (2) The highest yield was recorded at 25 °C, a salinity of 20, and at a CO_2_ of 700 ppm, indicating a synergistic effect of warming and acidification on biomass accumulation. (3) Peak cell permeability, used as a proxy for ichthyotoxicity in the absence of a known analyte, was observed under the most physiologically stressful conditions (15 °C, lower salinity water, and low CO_2_), highlighting the concern of this species in developing cooler, fresher waters.

While future ocean conditions may enhance the growth and biomass yield of *H. akashiwo*, they may concurrently reduce cell permeability and the associated ichthyotoxic potential. However, these conclusions are based on a single strain of *H. akashiwo*, and the species is known to exhibit strain-specific variability in ichthyotoxicity. Therefore, caution is warranted when generalizing these results. Future research should include additional strains and incorporate other stressors, such as nutrient enrichment and light variability.

Overall, this study contributes to our predictive understanding of how climate change may reshape harmful algal species’ distribution, productivity, and ichthyotoxicity in coastal ecosystems.

## 5. Materials and Methods

### 5.1. Culture of Microalgae

A unialgal strain of *H. akashiwo* (NWFSC-513), isolated initially from Clam Bay, WA, USA, in 2010, was cultured in f/2 medium (minus silicate) prepared with artificial seawater (ESAW) [67]. Cultures were maintained in 250 mL Erlenmeyer flasks at 20 ± 1 °C and under continuous illumination (80 ± 5 μmol photons m^−2^ s^−1^).

### 5.2. Experimental Conditions

Glassware was acid-washed in 1% HCl overnight and rinsed with ultrapure water. All experimental conditions were tested in triplicate to ensure reproducibility. Salinity was adjusted to 10, 20, or 30 by dissolving NaCl (Sigma-Aldrich, Oakville, ON, Canada) into ESAW. Before each experiment, *H. akashiwo* was acclimated to the designated salinity and grown to the mid-exponential phase (at Day 3–4 during the exponential growth phase). Cultures were diluted to 10,000 cells mL⁻^1^ in 50 mL Pyrex tubes (Corning, Corning, NY, USA), each sealed with a silicone stopper equipped with ports for gas, sampling, and pressure release.

Temperature control was achieved using refrigerated/heating circulators (VWR, Mississauga, ON, Canada), with temperature monitored three times daily using a Traceable™ Waterproof Thermometer (Fisher Scientific™, Ottawa, ON, Canada). Light intensity was maintained at 250 ± 10 μmol photons m⁻^2^ s⁻^1^ using a Quantum Scalar Laboratory sensor (Biospherical Instruments Inc., San Diego, CA, USA). CO_2_ (Praxair Canada Inc., London, ON, Canada) was filtered (0.45 μm) and bubbled into cultures for five minutes daily to maintain target concentrations, minimizing shear stress due to the delicate cell-wall-lacking morphology of *H. akashiwo* [43].

### 5.3. Growth Measurements

Cell density was assessed every 24 ± 1 h using a 0.5 mL aliquot, from which a 30 μL subsample was analyzed via flow cytometry (Turner Designs PhytoCyt flow cytometer (Sunnyvale, CA, USA)) using CFlow^®^ Plus software, version 1.0.227.5. Cells were gated using forward scatter and chlorophyll-a fluorescence, and density was calculated as:

(1) $$cell\;density=\frac{g}{v}\times 1000$$

*g* is the gated count and *v* is the sample volume (μL).

The specific growth rate (*K_e_*) during the exponential phase was calculated using [68].

(2) $$\;\;K_{e}=\frac{\ln(N_{t}-N_{0})}{t_{t}-t_{0}}$$

where *N*_0_ and *N_t_* are the cell concentrations (cells mL^−1^) over the (*t_t_* − *t*_0_) period.

The doublings per day (*k*) was computed as:

(3) $$\;\;\;k=\frac{K_{e}}{0.6931}$$

The yield was defined as the mean of the three highest cell densities measured at the end of the exponential or early stationary phases.

### 5.4. Cell Permeability Assay

Membrane permeability was quantified using SYTOX^®^ Green (Life Technologies, Carlsbad, CA, USA), which binds nucleic acids in membrane-compromised cells [69]. A 50 μM stock solution was stored at −20 °C. Background fluorescence was measured using Lugol’s iodine (0.5% *v*/*v*), and 30 μL samples were analyzed by flow cytometry (Ex: 488 nm, Em: 523 nm). For the test samples, 0.6 μM SYTOX^®^ Green was added and incubated in the dark for 15 min before measurement. Permeability was expressed as relative fluorescence units (RFU) after background subtraction. This approach offered a reliable proxy for evaluating membrane damage, which is often linked to ichthyotoxin release in *H. akashiwo* and other harmful algal species.

### 5.5. Design of Experiments (DOE)

A two-level complete factorial design (FFD) was used to assess the effects of temperature, salinity, and CO_2_ concentration, three key environmental stressors shaped by climate change, on growth, yield, and permeability. High (+1), low (−1), and center (0) coded values were assigned for each factor (Table 8). The selected ranges reflected values observed in natural aquatic environments as well as projected future conditions, ensuring their ecological relevance and realism.

Following FFD screening, response surface methodology (RSM) was used to explore the optimal conditions using a second-order polynomial model:

(4) $$Y=\beta_{0}+\sum_{i=1}^{k}\beta_{i}x_{i}+\sum_{i=1}^{k}\beta_{ii}x_{i}^{2}+\sum_{1\leq i\leq j}^{k}\beta_{ij}x_{i}x_{j}+\varepsilon$$

where *β*_0_ is the constant parameter; *k* is the number of variables; *x_i_* and *x_j_* are the design variables in coded values; and *β_i_*, *β_ii_*, and *β_ij_* are the coefficients of linear parameters, coefficients of quadratic parameters, and interaction parameters, respectively.

### 5.6. Statistical Analysis

Design Expert software (v10.0.3.1, Stat-Ease, Inc., Minneapolis, MS, USA) was used to create and analyze the experimental data and to conduct analysis of variance (ANOVA) or experimental design and model fitting. Statistical significance was determined at *p* < 0.05. All samples and experiments were performed in triplicate. The data were presented as a mean value ± standard deviation.

## Figures and Tables

**Table 1:** Experimental design for temperature, salinity, and CO_2_ levels and their effects on the growth responses of *H. akashiwo* isolated from Calm Bay, WA, USA (*n* = 3 ± standard deviation).

Run Order	Coded Values of Factors			Responses	
	Temperature (°C) X_1_	Salinity X_2_	CO_2_ Level (ppm) X_3_	Specific Growth Rate (K_e_) (d^−1^)	Doublings per Day (k) (d^−1^)
1	−1	−1	−1	0.17 ± 0.05	0.24 ± 0.08
2	+1	+1	+1	0.5 ± 0.05	0.71 ± 0.08
3	−1	−1	+1	0.12 ± 0.04	0.17 ± 0.06
4	−1	+1	−1	0.16 ± 0.04	0.24 ± 0.05
5	0	0	0	0.36 ± 0.05	0.52 ± 0.08
6	−1	+1	+1	0.19 ± 0.04	0.27 ± 0.06
7	+1	−1	+1	0.3 ± 0.10	0.43 ± 0.14
8	0	0	0	0.35 ± 0.08	0.51 ± 0.12
9	+1	+1	−1	0.78 ± 0.23	1.13 ± 0.33
10	+1	−1	−1	0.79 ± 0.15	1.13 ± 0.22
11	0	0	0	0.36 ± 0.05	0.52 ± 0.08
12	−1	0	0	0.17 ± 0.03	0.25 ± 0.05
13	0	0	0	0.4 ± 0.10	0.57 ± 0.14
14	0	−1	0	0.29 ± 0.04	0.42 ± 0.05
15	0	0	0	0.45 ± 0.07	0.65 ± 0.10
16	0	0	−1	0.45 ± 0.12	0.64 ± 0.17
17	0	0	+1	0.45 ± 0.10	0.65 ± 0.14
18	+1	0	0	0.59 ± 0.05	0.85 ± 0.08
19	0	+1	0	0.38 ± 0.05	0.55 ± 0.08

**Table 2:** ANOVA results for specific growth rate (K_e_) and doublings per day (k) of *H. akashiwo*.

Source		Remark	Sum of Squares	df	Mean Square	*F*-Value	*p*-Value Prob > *F*
Model	K_e_	Significant	0.61	6	0.10	31.76	<0.0001
	k	Significant	1.28	6	0.21	31.76	<0.0001
X_1_	K_e_	Significant	0.46	1	0.46	141.18	<0.0001
	k	Significant	0.95	1	0.95	141.18	<0.0001
X_2_	K_e_		0.012	1	0.012	3.70	0.0808
	k		0.025	1	0.025	3.70	0.0808
X_3_	K_e_	Significant	0.064	1	0.064	19.87	0.0010
	k	Significant	0.13	1	0.013	19.87	0.0010
X_1_X_2_	K_e_		0.002	1	0.00	0.067	0.430
	k		0.004	1	0.004	0.67	0.4304
X_1_X_3_	K_e_	Significant	0.072	1	0.072	22.21	0.0006
	k	Significant	0.15	1	0.15	22.21	0.0006
X_2_X_3_	K_e_		0.009	1	0.009	2.94	0.1146
	k		0.020	1	0.020	2.94	0.1146
R-squared	K_e_						0.9454
	k						0.9454
Adj. R-squared	K_e_						0.9157
	k						0.9157
Adeq precision	K_e_						17.800
	k						17.80

**Table 3:** The optimum factor set for the maximum growth rate and doublings per day of *H. akashiwo* is based on the Design of Experiments using the response surface methodology (DOE–RSM) approach.

Factors			Specific Growth Rate (K_e_) (d^−1^)		Doublings per Day (k) (d^−1^)	
Temperature (°C)	Salinity	CO_2_ Level	Predicted	Experimental	Predicted	Experimental
25	30	400	0.79 ± 0.06	0.78 ± 0.15	1.14 ± 0.08	1.13 ± 0.22

**Table 4:** Experimental design for temperature, salinity, and CO_2_ levels and their yield production for *H. akashiwo* isolated from Calm Bay, WA, USA (*n* = 3 ± standard deviation).

Run Order	Coded Values of Factors			Response
	Temperature (°C)	Salinity	CO_2_ Level (ppm)	Yield (Cells mL^−1^) (×10^3^)
1	−1	−1	−1	9.23 ± 1.03
2	+1	+1	+1	23.06 ± 2.06
3	−1	−1	+1	13.74 ± 2.74
4	−1	+1	−1	14.54 ± 1.25
5	0	0	0	20.58 ± 3.37
6	−1	+1	+1	20.49 ± 2.96
7	+1	−1	+1	23.39 ± 2.43
8	0	0	0	22.41 ± 1.72
9	+1	+1	−1	21.48 ± 2.46
10	+1	−1	−1	23.59 ± 1.96
11	0	0	0	21.46 ± 3.27
12	−1	0	0	15.33 ± 4.94
13	0	0	0	25.67 ± 4.80
14	0	−1	0	14.76 ± 1.79
15	0	0	0	23.37 ± 2.78
16	0	0	−1	21.69 ± 2.18
17	0	0	+1	21.39 ± 1.94
18	+1	0	0	21.94 ± 1.74
19	0	+1	0	21.71 ± 1.54

**Table 6:** Experimental design for temperature, salinity, and CO_2_ levels and their cell permeability for *H. akashiwo* isolated from Calm Bay, WA, USA (*n* = 3 ± standard deviation).

Run Order	Coded Values of Factors			Response
	Temperature (°C)	Salinity	CO_2_ Level (ppm)	Cell Permeability (RFU) (×10^3^)
1	−1	−1	−1	3.8 × 10^3^ ± 1227
2	+1	+1	+1	396 ± 153.1
3	−1	−1	+1	762 ± 181.8
4	−1	+1	−1	516 ± 256.5
5	0	0	0	956 ± 4982.3
6	−1	+1	+1	238 ± 83
7	+1	−1	+1	2.3 × 10^3^ ± 1263.1
8	0	0	0	850 ± 661.4
9	+1	+1	−1	355 ± 103.6
10	+1	−1	−1	740 ± 234.9
11	0	0	0	803 ± 509.1
12	−1	0	0	550 ± 172.3
13	0	0	0	459 ± 194.4
14	0	−1	0	1.7 × 10^3^ ± 496.8
15	0	0	0	1072 ± 813.7
16	0	0	−1	806 ± 261.0
17	0	0	+1	1.2 × 10^3^ ± 475.7
18	+1	0	0	715 ± 267.6
19	0	+1	0	405 ± 141.3

**Table 7:** ANOVA results for cell permeability of *H. akashiwo*.

Source	Remark	Sum of Squares	df	Mean Square	*F*-Value	*p*-Value
Model	Significant	9.323 × 10^12^	6	1.554 × 10^12^	5.17	0.0093
X_1_		1.795 × 10^11^	1	1.795 × 10^11^	0.60	0.4559
X_2_	Significant	5.488 × 10^12^	1	5.488 × 10^12^	18.27	0.0013
X_3_		1.651 × 10^11^	1	1.651 × 10^11^	0.55	0.4740
X_1_X_2_		2.797 × 10^11^	1	2.797 × 10^11^	0.93	0.3553
X_1_X_3_	Significant	3.026 × 10^12^	1	3.026 × 10^12^	10.07	0.0089
X_2_X_3_		1.857 × 10^11^	1	1.857 × 10^11^	0.62	0.4483
R-squared						0.7383
Adj. R-squared						0.5956
Adeq precision						9.398

**Table 8:** Experimental ranges and levels of the factors used in the factorial design.

Factor	Coded Symbol	Values of Coded Levels		
		−1	0	+1
Temperature (°C)	X_1_	15	20	25
Salinity	X_2_	10	20	30
CO_2_ level (ppm)	X_3_	400	550	700

## Data Availability

There are no supporting data for this paper.

## Abbreviations

The following abbreviations are used in this manuscript:

DOE	Design of Experiment
OFAT	One-Factor-at-a-Time
ANOVA	Analysis of Variance
2FI	Two-factor interaction
RSM	Response Surface Method
FFD	Full Factorial Design
HABs	Harmful Algal Blooms
ESAW	Enriched Artificial Seawater
